# Mitogenomic phylogeny of the common long-tailed macaque (*Macaca fascicularis fascicularis*)

**DOI:** 10.1186/s12864-015-1437-0

**Published:** 2015-03-21

**Authors:** Rasmus Liedigk, Jakob Kolleck, Kai O Böker, Erik Meijaard, Badrul Munir Md-Zain, Muhammad Abu Bakar Abdul-Latiff, Ahmad Ampeng, Maklarin Lakim, Pazil Abdul-Patah, Anthony J Tosi, Markus Brameier, Dietmar Zinner, Christian Roos

**Affiliations:** Primate Genetics Laboratory, German Primate Center, Leibniz Institute for Primate Research, Kellnerweg 4, 37077 Göttingen, Germany; Junior Research Group Medical RNA Biology, Primate Genetics Laboratory, German Primate Center, Leibniz Institute for Primate Research, Kellnerweg 4, 37077 Göttingen, Germany; Borneo Futures Project, People & Nature Consulting International, Country Woods house 306, JL. WR Supratman, Pondok Ranji, Ciputat, 15412 Jakarta, Indonesia; School of Archaeology & Anthropology, Building 14, Australian National University, Canberra, ACT 0200 Australia; School of Biological Sciences, University of Queensland, St. Lucia, QLD 4072 Australia; School of Environmental and Natural Resource Sciences, Faculty of Science and Technology, Universiti Kebangsaan Malaysia, 43600 Bangi, Selangor Malaysia; Sarawak Forest Department Hq, Wisma Sumber Alam Jalan Stadium, 93660 Petra Jaya Kuching, Sarawak Malaysia; Sabah Parks, Research and Education Division, PO Box 10626, 88806 Kota Kinabalu, Sabah Malaysia; Department of Wildlife and National Parks, Km 10, Jalan Cheras, 50664 Kuala Lumpur, Malaysia; Department of Anthropology, Kent State University, 238 Lowry Hall, Kent, OH 44242 USA; Cognitive Ethology Laboratory, German Primate Center, Leibniz Institute for Primate Research, Kellnerweg 4, 37077 Göttingen, Germany; Gene Bank of Primates, German Primate Center, Leibniz Institute for Primate Research, Kellnerweg 4, 37077 Göttingen, Germany

**Keywords:** Southeast Asia, Sundaland, Sanger sequencing, High-throughput sequencing, DNA-capture

## Abstract

**Background:**

Long-tailed macaques (*Macaca fascicularis*) are an important model species in biomedical research and reliable knowledge about their evolutionary history is essential for biomedical inferences. Ten subspecies have been recognized, of which most are restricted to small islands of Southeast Asia. In contrast, the common long-tailed macaque (*M. f. fascicularis*) is distributed over large parts of the Southeast Asian mainland and the Sundaland region. To shed more light on the phylogeny of *M. f. fascicularis*, we sequenced complete mitochondrial (mtDNA) genomes of 40 individuals from all over the taxon’s range, either by classical PCR-amplification and Sanger sequencing or by DNA-capture and high-throughput sequencing.

**Results:**

Both laboratory approaches yielded complete mtDNA genomes from *M. f. fascicularis* with high accuracy and/or coverage. According to our phylogenetic reconstructions, *M. f. fascicularis* initially diverged into two clades 1.70 million years ago (Ma), with one including haplotypes from mainland Southeast Asia, the Malay Peninsula and North Sumatra (Clade A) and the other, haplotypes from the islands of Bangka, Java, Borneo, Timor, and the Philippines (Clade B). The three geographical populations of Clade A appear as paraphyletic groups, while local populations of Clade B form monophyletic clades with the exception of a Philippine individual which is nested within the Borneo clade. Further, in Clade B the branching pattern among main clades/lineages remains largely unresolved, most likely due to their relatively rapid diversification 0.93-0.84 Ma.

**Conclusions:**

Both laboratory methods have proven to be powerful to generate complete mtDNA genome data with similarly high accuracy, with the DNA-capture and high-throughput sequencing approach as the most promising and only practical option to obtain such data from highly degraded DNA, in time and with relatively low costs. The application of complete mtDNA genomes yields new insights into the evolutionary history of *M. f. fascicularis* by providing a more robust phylogeny and more reliable divergence age estimations than earlier studies.

**Electronic supplementary material:**

The online version of this article (doi:10.1186/s12864-015-1437-0) contains supplementary material, which is available to authorized users.

## Background

Macaques (genus *Macaca*) represent one of the most successful extant primate radiations. They colonized a large geographic range, from continents to islands, making them unique among non-human primates [1]. Fossils indicate that they arose in northern Africa around 7 million years ago (Ma) [2]. During their expansion into Asia in the late Miocene, the genus diversified into various species groups and species that have been defined by morphological, behavioral and molecular characters, and by their geographic distribution [2-9].

Taxonomic and phylogenetic affiliations of the various macaque species have been matter of debate for several decades [1-7,10]. According to current classifications the genus comprises 22 species, which are divided into seven species groups [9,11]; among them three monotypic species groups, (1) the *M. sylvanus* group, (2) the *M. arctoides* group and (3) the *M. fascicularis* group, and four polytypic groups, (4) the Sulawesi macaques group with six species, (5) the *M. mulatta* group with three species, (6) the *M. sinica* group with five species and (7) the *M. silenus* group with five species. As with the other species groups, the species composition of the *M. fascicularis* group has changed over time. Delson [2] and Fooden [3,4] included four species (*M. mulatta*, *M. cyclopis*, *M. fuscata*, *M. fascicularis*), but Groves [6] moved *M. mulatta*, *M. cyclopis* and *M. fuscata* in their own species group, the *M. mulatta* group, and integrated *M. arctoides* in the *M. fascicularis* group. Zinner et al. [9] likewise recognized the members of the *M. mulatta* group as a distinct species group and additionally excluded *M. arctoides* proposing a monotypic *M. fascicularis* group.

**Figure 1:**
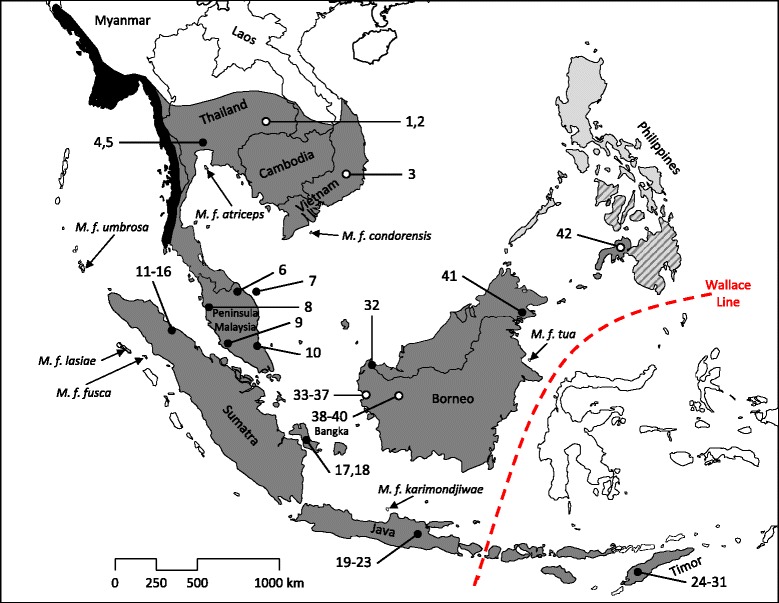
**Geographical distribution of long-tailed macaques (** ***Macaca fascicularis*** **) and geographical origin of samples.** Species and subspecies distributions are depicted according to [12] and adapted from [15]. The distribution of *M. f. aureus*, *M. f. fascicularis* and *M. f. philippinensis* is indicated by black, dark grey and light grey regions, respectively; the hatched region indicates the transition zone between the latter two subspecies. Subspecies on small islands are named in the map as are countries and islands mentioned in the text. Open and filled circles indicate approximate and exact geographical origin of *M. f. fascicularis* samples, respectively. ID numbers correspond to those in Figure 2 and Additional file 2: Table S2.

The long-tailed macaque (*M. fascicularis*) has the most discontinuous, and beside rhesus macaques, the largest distribution of all macaque species. Its range covers the southern part of the Southeast Asian mainland (Bangladesh, Myanmar, Thailand, Laos, Vietnam, Cambodia, peninsular Malaysia) as well as most of Sundaland (the islands of Borneo, Sumatra and Java, and adjacent islands) and beyond (islands east of the Wallace Line, Philippines) (Figure 1). On the basis of differences in pelage coloration and tail length ten subspecies are currently recognized [6,8,9,11-15]. Three of them (*M. f. aureus*, *M. f. fascicularis*, *M. f. philippinensis*) have relatively large distributions, while all others (*M. f. atriceps*, *M. f. condorensis*, *M. f. fuscus*, *M. f. karimondjawae*, *M. f. lasiae*, *M. f. tua*, *M. f. umbrosus*) are restricted to small islands (Figure 1). However, genetic data are not yet available for a comparative assessment of this classification; so far, genetic studies have included only samples from *M. f. fascicularis* and *M. f. philippinensis*. Given the large and discontinuous range of *M. f. fascicularis*, it is not surprising that (genetic) variation within this subspecies is high [16-26]. In fact, there is a deep genetic differentiation between *M. f. fascicularis* from the Asian mainland and from Sundaland [16-21,23-26]. Interestingly, on Sumatra, Y-chromosomal lineages from both, mainland and Sundaland are found [19]. Besides the genetic variation among local long-tailed macaque populations, molecular studies have also revealed significant gene flow from rhesus macaques (*M. mulatta*) into Indochinese *M. f. fascicularis* [17,27-29]. Recent genome data indicate that around 30% of the Asian mainland *M. fascicularis* genome is of rhesus macaque origin [30]. This ancient hybridization (gene flow) most likely occurred unidirectional (male-mediated), from rhesus into long-tailed macaques and not vice versa, i.e. analyses of maternally inherited markers such as mitochondria will not resolve the question of hybridization [17,27,28,30]. Even today, hybridization between both species occurs in a wide hybrid zone running from Vietnam, through Laos, Thailand, and probably into Myanmar [13,31]. Since the long-tailed macaque is an important model organism in biomedical research, reliable knowledge about its evolutionary history and genetic composition is essential for biomedical inferences, particularly since local populations show extreme genetic, physiological and behavioral variation (for an overview see [32]).

The geographic origin of *M. fascicularis* and dispersal scenarios that led to its current distribution are still a matter of debate. Delson [2] suggested that macaques entered Sundaland, probably in the Pliocene, during periods of low sea level and ancestral *M. fascicularis* became isolated there when rising sea levels and geological activity fragmented Sundaland. During the Pleistocene, *M. fascicularis* extended its range again [2,14]. This largely corresponds to the observed higher level of nucleotide diversity found in long-tailed macaque populations from Sundaland compared to the populations from the Asian mainland and Malay Peninsula [18,23]. This scenario is also in agreement with the fact that the earliest fossils of *M. fascicularis*, or at least those of a close relative, were found on Java [2,14,33]. Currently, the species is also found on islands that were never connected to the Asian mainland or Sundaland, including islands beyond the Wallace line (e.g., Lombok, Sumbawa, Flores, Timor) and the Philippines. Accordingly, it was assumed that humans introduced *M. fascicularis* to the islands east of the Wallace line ca. 4,000 year ago [14], while the Philippines were most likely naturally colonized during two independent immigration events [26]. The species’ survival in areas, where it has been introduced by humans (e.g., Hong Kong, Taiwan, Papua New Guinea, New Britain, and various Pacific islands), indicates its significant ecological plasticity. Long-tailed macaques are naturally adapted to riverine and coastal environments such as mangrove and gallery forests [34,35]. They primarily feed on fruits and seeds [36], but as indicated by one of its common names, crab-eating macaque, they also include crabs, shrimps, clams and fish in their diet [37]. They are able to swim and even to dive [38]. Hence, it is likely that long-tailed macaques were able to cross short distances between islands actively.

The objective of this study is to shed more light on the phylogeny of *M. f. fascicularis*, the most widespread subspecies of the long-tailed macaques, occurring on the Southeast Asian mainland and Sundaland islands, including parts of the Philippines and east as far as Timor. Therefore, we generated complete mitochondrial (mtDNA) genomes from 40 long-tailed macaque individuals either by traditional polymerase chain reaction (PCR) amplification followed by Sanger sequencing or by DNA-capture and high-throughput sequencing. We expect that the analysis of complete mtDNA genomes provides a better resolution of phylogenetic relationships among lineages than only short mtDNA fragments.

## Results

We generated 42 complete mtDNA genome sequences from 40 *M. fascicularis* individuals, either by classical PCR followed by Sanger sequencing (10 individuals) or by DNA-capture and high-throughput sequencing (32 individuals). For two museum samples (IDs: 20, 31) both methods were applied which yielded sequences with 100% identity, thus indicating similarly high accuracy of both methods. For mtDNA genomes that were captured and sequenced on the Ion PGM sequencing platform we obtained an average of 97,583 (12,599-230,683) trimmed reads with an average read length of 96 bp, resulting in an average 285-fold coverage. Sequences in the overlapping parts were identical and all protein-coding genes were correctly translated without any premature stop codons, indicating that no nuclear mitochondrial-like sequences (numts) are present in our dataset. All newly generated mtDNA genomes had a length of 16,561 to 16,567 bp, and consisted of 22 transfer RNA genes, 2 ribosomal RNA genes, 13 protein coding genes and the control region.

**Figure 2:**
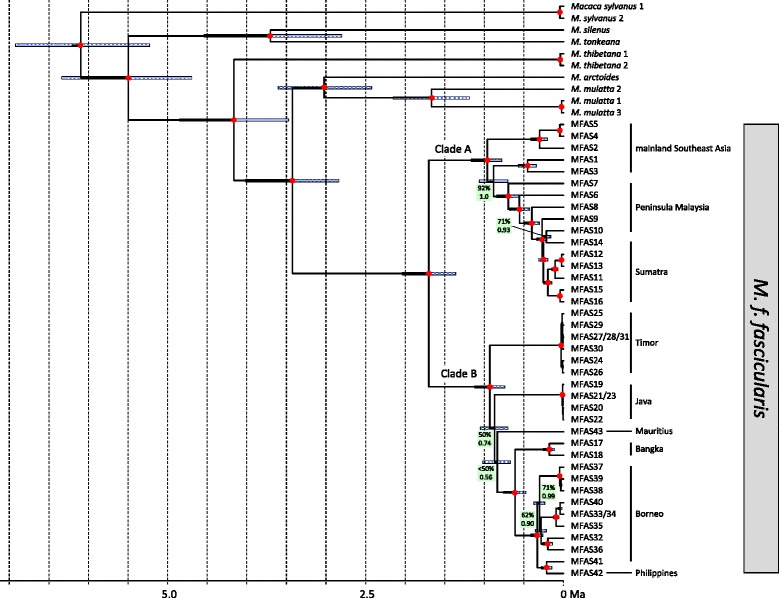
**Ultrametric tree showing phylogenetic relationships and divergence ages among macaques as calculated from complete mtDNA genome sequences (relationships among non-macaque taxa not shown).** Red dots indicate ML bootstrap support of > 95% and Bayesian posterior probabilities of 1.0; values below are given at respective nodes. Blue bars indicate 95% CIs of divergence times and the time scale below shows million years before present. IDs correspond to those in Figure 1 and Additional file 2: Table S2. For detailed information about divergence ages and 95% CIs see Additional file 1: Table S1.

For phylogenetic analysis, we generated an alignment including the 40 newly generated and 20 additional mtDNA genomes downloaded from Genbank. The resulting alignment had a length of 16,874 bp, but was reduced to 15,868 bp after indels and poorly aligned positions were removed. Some *M. f. fascicularis* individuals shared the same haplotype (IDs: 21 = 23, 27 = 28 = 31, 33 = 34). These were excluded resulting in a final alignment of 56 unique primate mtDNA genome haplotypes. Both alignments are available for download (Supplementary data [39]). Phylogenetic trees as obtained from maximum-likelihood (ML) and Bayesian analyses are nearly identical and most nodes are strongly supported (ML bootstrap values: > 95%, Bayesian posterior probabilities: 1.0; Figure 2). According to estimated divergence ages, Hominidae and Cercopithecidae separated 28.60 (95% credibility interval [CI]: 25.31-31.78) million years ago (Ma) (for estimates and their 95% CIs see Figure 2 and Additional file 1: Table S1). Among hominids, *Pongo* diverged from the *Homo* + *Pan* clade 13.82 (12.68-14.86) Ma, while the latter split 6.32 (5.73-6.89) Ma. Among cercopithecids, *Colobus* diverged first, 19.89 (16.17-23.87) Ma, and *Chlorocebus* separated from papionins 12.81 (10.59-15.22) Ma. In the Papionini clade, *Theropithecus* and *Papio* diverged from macaques 10.90 (8.92-12.90) Ma, while the former two genera split 4.77 (3.87-5.72) Ma. Within macaques, the African *M. sylvanus* branched off first, 6.10 (5.23-6.92) Ma. The remaining, solely Asian macaque species, diverged into two clades 5.49 (4.69-6.34) Ma, one comprising *M. silenus* and *M. tonkeana*, and the other *M. thibetana*, *M. arctoides*, *M. mulatta* and *M. fascicularis*. In the former clade, *M. silenus* and *M. tonkeana* separated 3.70 (2.80-4.54) Ma, while in the latter clade *M. thibetana* split off first, 4.16 (3.47-4.85) Ma, followed by *M. fascicularis* 3.42 (2.83-4.01) Ma, before finally *M. mulatta* and *M. arctoides* diverged 3.02 (2.42-3.60) Ma. Within *M. fascicularis*, an initial split occurred 1.70 (1.36-2.04) Ma, separating haplotypes from mainland Southeast Asia, Peninsula Malaysia and North Sumatra (Clade A), and haplotypes from Borneo, Java, Bangka, Timor, the Philippines and Mauritius (Clade B). In Clade A, individuals from mainland Southeast Asia, Peninsula Malaysia and North Sumatra do not form reciprocally monophyletic clades. Splitting events in Clade A started 0.96 (0.78-1.16) Ma. In Clade B, individuals from different geographic regions form monophyletic clades or represent distinct lineages. The only exception is the Borneo clade, which also includes the individual from the Philippines (ID: 42). In clade B, the branching pattern among main clades/lineages remains unresolved (ML bootstrap values: <50-50%, Bayesian posterior probabilities: 0.56-0.74) indicating a diversification within a short time period. In fact, this radiation was estimated to have occurred between 0.93 Ma (0.74-1.12) and 0.84 (0.67-1.02) Ma, thus in less than 100,000 years. Individuals from Bangka (IDs: 17, 18), an island east of Sumatra (Figure 1), form a monophyletic clade and separated from the Borneo/Philippines clade 0.61 (0.47-0.75) Ma. The Philippine individual is nested within the Borneo clade and specifically clusters with an individual from Sabah (ID: 41); they diverged from each other 0.21 (0.15-0.28) Ma.

## Discussion

By applying different methods, classical PCR amplification followed by Sanger sequencing and DNA-capture with subsequent high-throughput sequencing, we successfully obtained complete mtDNA genome data from 40 *M. fascicularis* individuals. Both methods have proven to be useful to gain such data with similarly high accuracy, but the DNA-capture and high-throughput sequencing approach is less costly and time consuming [40-42]. Moreover, DNA extracted from museum material and fecal material is normally highly degraded. Fortunately, some of our museum and fecal samples contained DNA in sufficient quality so that the complete mtDNA genome could be amplified via 21 overlapping PCRs. Usually, due to the high degree of DNA degradation, generating complete mtDNA genomes would require a much larger number of PCR amplifications. In contrast, DNA-capture does not need a certain DNA fragment size, because any size of DNA fragment can be captured and subsequently sequenced. However, degraded DNA should be solely investigated in special laboratories and with various precautions to prevent contamination.

Our results concerning the phylogenetic relationships among macaque and non-macaque taxa and estimated divergence ages are largely in line with previous molecular studies [5,7,10,19,43-48]. For the phylogenetic relationships among *M. fascicularis* haplotypes, we obtained higher statistical support for most nodes in our tree, as compared to earlier mtDNA studies which used only fragments of the mtDNA genome [5,7,17-20,22]. Nevertheless, some nodes in our study are still missing significant statistical support, thus leaving some phylogenetic relationships, in particular those between populations from Timor, Java, Mauritius and Bangka/Borneo/Philippines, unresolved. Such results are common when clades or lineages diverged within a short time period [42,48-52].

In contrast to Tosi and Coke [19] who found Sumatran individuals to be part of the Sundaland clade (referring to our Clade B), the Sumatran individuals, which we analyzed, are nested within the Asian mainland clade (referring to our Clade A). A possible explanation for this contradiction is the different geographic origin of studied individuals, with the South Sumatran samples of Tosi and Coke [19] clustering with Sundaland sequences and our North Sumatran samples clustering with mainland sequences. Thus, both major *M. f. fascicularis* mtDNA clades are likely to be present on Sumatra similar to the presence of both Y chromosomal haplogroups on the island [19].

Since mtDNA is only inherited via the maternal line and macaques live mainly in female philopatric societies [53,54], mtDNA data can be utilized to reveal insights into genetic differences among regional populations and to trace their phylogeographic history [55]. According to our phylogenetic reconstruction and estimated divergence ages, *M. f. fascicularis* initially split into two clades 1.70 (1.36-2.04) Ma, with representatives of both lineages being found today on Sumatra (according to our study and [19]). Possible explanations therefore are (1) Sumatra is the place of origin of *M. f. fascicularis*, (2) Sumatra is the place of origin of only Sundaland *M. f. fascicularis*, while long-tailed macaques from the mainland invaded the island later, or (3) long-tailed macaques on Sumatra became extinct and the island was later re-colonized from the mainland and other Sundaland islands. The hypothesis that Sumatra is the place of *M. f. fascicularis* origin is supported by the observed high mtDNA diversity found on the island compared to other regions where the subspecies occurs (e.g., [26]). However, not in support of this hypothesis is the paraphyly of haplotypes from the mainland and Malay Peninsula, and the respective branching pattern among them and the Sumatra haplotypes, which suggests that the northern Sumatra population came in from the mainland. To test whether Sumatra or any other island, e.g., Java [2,18] is the place of origin of Sundaland or all *M. f. fascicularis* populations, needs further investigations and, particularly, should include data of *M. f. fascicularis* from southern Sumatra.

As in previous studies [7,19,26], we found long-tailed macaques from the Philippines clustering within the Borneo clade. Since the Bornean individual, which is most closely related to the Philippine specimens, is from the furthest east of Borneo (Sabah, Tawau Hill Park), this branching pattern fosters the previously proposed hypothesis of a colonization of the Philippines via Borneo [1,26]. Within the last million years, the Philippines have never been connected to the Southeast Asian mainland or Borneo via a continuous land bridge [56]. One possible exception is the island of Palawan, which has been considered to have had a land connection to Borneo during sea level low-stands in the late Pleistocene [57]. The previously proposed Philippine colonization hypothesis [1,26] via Palawan and appending islets seems plausible (stepping-stone colonization). A recent study suggests that there may have been at least two dispersal events from Borneo into the Philippines, first one via Palawan resulting in *M. f. philippinensis* found in the north of the Philippine Archipelago (Figure 1), and a later one via the Sulu Archipelago that resulted in *M. f. fascicularis* in the south [26]. Our Philippine sample most likely belongs to this southern taxon.

One noteworthy outcome from our study is the early divergence of a monophyletic Timor clade within the Sundaland clade (Figure 2). It appears that this clade diverged some 0.93 (1.12-0.74) Ma from the other Sundaland lineages. This finding is supported by an analysis of blood protein polymorphisms from samples across the Indonesian and Timor island arc, indicating that populations east of the Wallace Line (Lombok and Sumbawa) have greatly differentiated from those to the west [58]. Our mtDNA-based estimate, however, significantly predates the earliest finds of macaques in Timor’s archaeological record, which appear at the same time, i.e. as the first evidence of pottery and domesticated pig in one site a few thousand years ago, indicating human translocations [59]. Similarly, on Flores, an island further west, but still east of the Wallace Line, long-tailed macaques only appear in the archaeological record around 7,000 years ago [60]. It is unclear what underlies the apparent major discrepancy between the present phylogenetic analysis and the zooarchaeological record, but an introduction by humans as proposed by Fooden [14] seems unlikely, although the possibility remains that the detected Timor haplotypes originated from somewhere else in Sundaland, a place that was not sampled in our study.

## Conclusions

Both applied laboratory methods have proven to be powerful to generate complete mtDNA genome data with similarly high accuracy, with the DNA-capture and high-throughput sequencing approach as the most promising and only practical option to obtain such data from highly degraded DNA, fast and relatively cheap. Our study provides new insights into the evolutionary history of *M. f. fascicularis*, most prominent we obtained first evidence for the presence of haplotypes in North Sumatra that are related to Asian mainland haplotypes and the clearly distinct and phylogenetically old Timor clade. However, to fully resolve the phylogeny of long-tailed macaques, to identify their origin and the dispersal routes leading to their current distribution, to assess their full genetic diversity and to explore to which extent secondary gene flow occurred between local populations, it is fundamental to include further *M. f. fascicularis* populations from throughout their range into future studies. In these studies both, mitochondrial and a large number of nuclear loci, should be analyzed. Moreover, to fully understand the evolutionary history of the species, the other subspecies of *M. fascicularis* should be incorporated in such studies as well. Since long-tailed macaques are an important model species in biomedical research and considering intra-specific variation in genetics, physiology and behavior, more attention should be paid to the selection of study specimens.

## Methods

### Ethical statement

Blood samples were taken during routine health checks by experienced veterinarians and not specifically for this study. All research complied with protocols approved by the Animal Welfare Body of the DPZ in Germany and the Department of Wildlife and National Parks in Malaysia, and adhered to the legal requirements of the countries, in which research was conducted. The study was carried out in compliance with the principles of the American Society of Primatologists for the ethical treatment of non-human primates (https://www.asp.org/society/resolutions/EthicalTreatmentOfNonHumanPrimates.cfm). No animals were sacrificed for this study.

### Sample collection

We collected and sequenced mtDNA genomes from 40 long-tailed macaque individuals originating from 16 sites throughout the species’ range in Southeast Asia and Sundaland, and from the introduced population on Mauritius (Figure 1, Additional file 2: Table S2). Thirty-one of our samples (sample IDs: 4, 5, 11–31, 33–40) derived from museum specimens housed in the Bavarian State Collection of Zoology (ZSM) in Munich, Germany. Respective specimens were collected between 1904 and 1949. Dried muscle tissue attached to the skeleton was taken with sterilized scalpels and tweezers, and gloves and masks were worn during sample collection to avoid contamination. Museum samples were stored dry in tubes or plastic envelopes. Additionally, we included seven fresh fecal samples, stored in 90% ethanol, which were collected during field surveys (IDs: 6–10, 32, 41). We further obtained high-quality DNA extracted from blood samples from each one individual from Covance Inc. (Münster, Germany) and the German Primate Center (DPZ, Göttingen, Germany), which originated from the Philippines (ID: 42), and Mauritius (ID: 43), respectively. For all samples, we tried to obtain information about the exact geographic provenance, but this was not always possible. While for all fecal samples, GPS coordinates were recorded, information about the exact origin of the samples from the Philippines and Mauritius was not available. Likewise, we were not able to identify the exact provenance of five Bornean samples (IDs: 33–37, derived from “west coast Borneo”), while for all other museum samples the exact origin could be determined. Thus, 38 samples can be geographically clearly assigned to *M. f. fascicularis* (IDs: 4–41). The individual from Mauritius (ID: 43) most likely refers also to *M. f. fascicularis* because it is believed that this introduced population originated from Sumatra or at least from Sundaland [19,61], while the individual from the Philippines (ID: 42) could be either *M. f. philippinensis* or *M. f. fascicularis* (due to its haplotype it is most likely *M. f. fascicularis*). The individuals from Timor refer to the holotype (ID: 24) and paratypes (IDs: 25–31) of *Pithecus fascicularis limitis* Schwarz, 1913, which is recognized as synonym of *M. f. fascicularis* [6,12]. Beside the 40 samples mentioned above, we obtained a blood sample from an additional *M. f. fascicularis* individual (ID: 3) from Convance Inc. which originated from Vietnam. The mtDNA genome of this individual was already published [48], but its DNA was used to prepare baits for DNA-capture. For detailed sample information see Additional file 2: Table S2.

### DNA extraction

For the extraction of total genomic DNA we used two different methods. First, we applied a kit-based method using the First-DNA All Tissue kit (Gen-Ial). All fecal and five of the museum samples (IDs: 11, 14, 20, 31, 38) were extracted with this method following respective protocols provided by the company. To avoid and check for cross-sample contamination, all working steps were carried out in separate laboratories and under Captair Bio PCR cabinets (Erlab), gloves and masks were permanently worn, and negative extraction controls were routinely performed. Further, samples were treated one by one, and workbenches were decontaminated with UV light before and after each extraction. After extraction, DNA concentration was measured on a NanoDrop ND-1000 spectrophotometer and samples were stored at −20°C until further processing.

Secondly, 28 museum samples (IDs: 4, 5, 12, 13, 15–31, 33–37, 39, 40) were extracted in a special ancient DNA laboratory applying a protocol for nondestructive DNA extraction [62,63] with slight modifications [64]. All working steps were carried out in Thermo Scientific Safe 2020 biological safety cabinets. For each step (sample preparation, DNA extraction) different cabinets were used, and before and after each sample, cabinets were cleaned with DNA decontamination solution and treated with UV light for at least 30 min. Concentration of extracted DNAs was measured on a Qubit 2.0 fluorometer and DNA samples were frozen at −20°C until further processing. For comparative reasons, two museum samples (IDs: 20, 31) were extracted with both methods.

### DNA amplification and Sanger sequencing

We generated complete mtDNA genomes from the high-quality samples from the Philippines and Mauritius as well as from three of the fecal samples (IDs: 7, 32, 41) and five of the museum samples (IDs: 11, 14, 20, 31, 38) by traditional PCR amplification followed by Sanger sequencing. All working steps (PCR setup, gel electrophoresis, PCR product purification, sequencing) were conducted in separate laboratories and under Captair Bio PCR cabinets to prevent cross-sample contamination. Further, negative PCR controls (without template DNA) were routinely conducted. To minimize the risk of amplifying numts for the two high-quality DNA samples, we produced two overlapping long-range PCR products (8 kb and 10 kb) followed by 21 nested PCRs with product sizes of 1.0-1.2 kb and an overlap of 100–300 bp applying methods described elsewhere [48]. Since DNA extracted from fecal and museum samples is usually degraded, the complete mtDNA genome from these samples was directly amplified via the 21 PCRs mentioned above and not first via two long-range PCRs. PCR conditions were the same as for the nested PCRs above, but sometimes the number of cycles was increased to 60. As template, we added 10–50 ng DNA to the reaction. PCR performance and product sizes were checked on 1% agarose gels, and after purification, PCR products were sequenced on an ABI 3130*xL* sequencer using the BigDye Terminator Cycle Sequencing kit (Applied Biosystems) and both amplification primers. Information on primers and PCR conditions is available upon request. Sequences were checked with 4Peaks 1.7.1 (www.mekentosj.com) and mtDNA genomes were assembled with SeaView 4.4.0 [65]. Annotation was performed with DOGMA [66] and manually verified.

### DNA-capture and high-throughput sequencing

Complete mtDNA genomes from 28 museum (IDs: 4, 5, 12, 13, 15–31, 33–37, 39, 40) and four fecal samples (IDs: 6, 8–10) were generated using a DNA-capture approach followed by high-throughput sequencing according to Maricic et al. [40] with slight modifications (see below) to adapt the workflow to the Ion PGM sequencing system (Ion Torrent). To prevent contamination, all working steps were carried out in dedicated ancient DNA and/or special high-throughput sequencing laboratories, and various negative controls were applied. After DNA extraction and concentration measurement, barcoded sequencing libraries were established using the Ion Plus Fragment Library kit and the Ion Xpress Barcode Adapters. Adapter ligation and the subsequent amplification of the samples were performed according to the protocol for Ion Xpress Plus gDNA Fragment Library Preparation. Afterwards, we pooled the adapter-ligated and amplified libraries in equal concentrations to a total of 2 μg. As bait we used mtDNA genomes of each one long-tailed macaque individual from Vietnam (ID: 3) and Mauritius (ID: 43). The respective complete mtDNA genomes were amplified via two overlapping PCR products (see above). Afterwards, we sheared the PCR products to an average of ca. 1,000 bp fragments with a Bioruptor Pico. We diluted 1.5 μg of PCR product to a volume of 150 μl, split the sample into three (50 μl each) and sonicated each six times with 10 seconds “ON” and 90 seconds “OFF”. One μl of the sheared PCR product was size-checked on the Agilent 2100 Bioanalyzer with the high sensitivity DNA kit. Fragments were subsequently end-repaired, biotinylated by ligating the Bio-T/B adapter [40], and immobilized on streptavidin-coated beads. Bait and the pooled single-stranded libraries were combined and four phosphorylated blocking oligos (BO1.P1.F: CCACTACGCCTCCGCTTTCCTCTCTATGGGCAGTCGGTGAT-phosphate, BO2.P1.R: ATCACCGACTGCCCATAGAGAGGAAAGCGGAGGCGTAGTGG-phosphate, BO3.A.F: CCATCTCATCCCTGCGTGTCTCCGACTCAG-phosphate, BO4.A.R: CTGAGTCGGAGACACGCAGGGATGAGATGG-phosphate) were added. After 48 h of hybridization at 65°C, library molecules that did not hybridize were washed out and the enriched library pool was eluted. Subsequently, the concentration of the enriched library pool was measured by qPCR (Ion Library Quantitation Kit) and sequenced on the Ion PGM sequencer using a 316v2 or 318v2 chip and the Ion PGM Sequencing 400 Kit protocol. The raw sequencing reads were quality-filtered, and adapters and barcodes were trimmed with the PGM Torrent Suite Software 4.2. The extracted reads were initially assembled with the Newbler program (GS Reference Mapper) of the 454 Sequencing System Software 2.5 from command line with standard parameters. The mtDNA genome of the Vietnamese *M. f. fascicularis* individual (ID: 3) was used as reference. Batch processing was done by custom Perl scripts. The resulting contigs, typically ranging from 1 to 4 sequences per mtDNA genome, were manually assembled into genomes with SeaView and annotated with DOGMA. All gaps between contigs could be closed by combining the results from multiple sequencing runs.

### Statistical analyses

For phylogenetic reconstructions, we expanded our dataset with additional mtDNA genome sequences from macaque and non-macaque taxa derived from Genbank. The dataset comprised 60 mtDNA genomes including 43 *M. fascicularis* individuals (3 from Genbank including ID: 3), at least one representative of the other six macaque species groups (2 *M. sylvanus*, 1 *M. arctoides*, 3 *M. mulatta*, 2 *M. thibetana*, 1 *M. tonkeana*, 1 *M. silenus*) and various outgroup taxa (1 *Theropithecus gelada*, 1 *Papio hamadryas*, 1 *Chlorocebus pygerythrus*, 1 *Colobus guereza*, 1 *Pongo abelii*, 1 *Pan troglodytes*, 1 *Homo sapiens*). For detailed sample information and Genbank accession numbers see Additional file  2: Table S2.

Sequences were aligned with Muscle 3.7 [67] as implemented in SeaView and manually corrected. Indels and poorly align positions were removed with Gblocks 0.91b [68] using standard settings. Identical sequences were subsequently excluded (IDs: 21 = 23, 27 = 28 = 31, 33 = 34), resulting in a final dataset of 56 unique mtDNA genome haplotypes. For ML and Bayesian tree reconstructions, we applied the programs RAxML 0.93 [69] and MrBayes 3.1.2 [70,71], respectively. ML calculations in RAxML were run with the CAT-GTR model and 1,000 bootstrapping replications. For Bayesian tree reconstructions in MrBayes, we conducted four Markov Chain Monte Carlo (MCMC) runs with a default temperature of 0.2 and the TrN + I + G model as selected as best-fit model in jModeltest 2.1 [72] under the Bayesian information criterion (BIC) and the Decision Theory Performance-based Selection (DT). All repetitions were run for 1 million generations with tree and parameter sampling setting in every 100 generations. The first 25% of samples were discarded as burn-in, resulting in 75,001 trees per run. The adequacy of the burn-in and convergence of all parameters was assessed via the uncorrected potential scale reduction factor (PSRF) [73] as calculated by MrBayes and by visual inspection of the trace of the parameters across generations using TRACER 1.5 [74]. To check whether posterior clade probabilities were also converging, AWTY [75] was applied. Posterior probabilities for each split and a phylogram with mean branch lengths were calculated from the posterior density of trees.

Divergence ages from the dataset were estimated with BEAST 1.6.1 [76] applying a Bayesian MCMC method with a relaxed molecular clock approach [77]. A relaxed lognormal model of lineage variation and a Birth-Death Process prior for branching rates was assumed. The following five fossil-based calibration points were used with a normal distribution prior for respective nodes: (1) the *Homo* – *Pan* split 6.5 Ma with a 95% CI of 0.5 Ma [78-80], (2) the split between *Pongo* and the *Homo* + *Pan* clade at 14 Ma (95% CI: 1.0 Ma) [81], (3) the divergence of *Theropithecus* and *Papio* 5 Ma (95% CI: 1.5 Ma) [82,83], (4) the split between African (*M. sylvanus*) and Asian macaques at 5.5 Ma (95% CI: 1.0 Ma) [83,84] and (5) the divergence of hominids and cercopithecids at 27.5 Ma (95% CI: 3.5) [85-87]. Four replicates were run in BEAST for 25 million generations with tree and parameter sampling occurring every 100 generations. TRACER was used to assess the adequacy of a 10% burn-in and the convergence of all parameters via visual inspection of the trace of the parameter across generations. Sampling distributions were combined (25% burn-in) using the software LogCombiner 1.6.1. A consensus chronogram with node height distribution was generated and visualized with TreeAnnotator 1.6.1 and FigTree 1.4.0 [88].

### Availability of supporting data

The alignments supporting the results of this article are available in the Data Dryad repository, 10.5061/dryad.c801k.

## Additional files

Additional file 1: Table S1. Estimated divergence ages in Ma and 95% CIs (in parentheses) among lineages. Additional file 2: Table S2. Detailed information about studied mtDNA genomes (geographical origin, source, Genbank accession number, sequencing method).
